# Children and ADRs (Adverse Drug Reactions)

**DOI:** 10.1186/1824-7288-36-4

**Published:** 2010-01-15

**Authors:** Ettore Napoleone

**Affiliations:** 1 S.I.P (Italian Society of Paediatrics), Molise, Italy; 2 FIMP- MCRN (Family Pediatricians -Medicines for Children Research Network), Italy; 3 Pediatric Working Group AIFA (Italian Medicines Agency), Italy

## Abstract

Many medicines are prescribed to the paediatric population on an unlicensed or 'off-label' basis because they have not been adequately tested and/or formulated and authorized for use in appropriate paediatric age groups. Regulatory authorities also need to remind health professionals about the importance of their contribution towards the process of paediatric pharmacovigilance thanks to their reporting of adverse drug reactions.

The lack of reliable data in the paediatric population is associated with specific problems including: limited availability of safety data due to the lack of clinical trials in the paediatric population; under- or over-dosing in some age groups due to the lack of pharmacokinetics data or dose-finding studies; maturation, growth and development of the paediatric population susceptible to drug-induced growth and development disorders as well as to delayed ADRs not findable in adults.

Pre-marketing trials are able to provide information about the benefits of drugs but do not manage to establish a safety profile. Spontaneous reporting of suspected ADRs become an important means to promote reasonable warning signs.

Therefore some ADRs may be known in their qualitative aspect and quantitative aspect only after successful marketing and use in the population during a "normal" use. When the drug is used in clinical practice in large unselected populations, epidemiological post-marketing studies are useful as they find their major confirmation in recalling all the events that occur during monitoring, with estimates of incidence of ADRs that can not be obtained by spontaneous reports.

In these studies a significant role can be played by the Family Pediatricians with the participation to active pharmacovigilance projects.

## Introduction

Many medicines are prescribed to the paediatric population on an unlicensed or 'off-label' basis because they have not been adequately tested and/or formulated and authorized for use in appropriate paediatric age groups. Regulatory authorities also need to remind health professionals about the importance of their contribution towards the process of paediatric pharmacovigilance thanks to their reporting of adverse drug reactions (ADRs). The practice of pharmacovigilance for paediatric use medicines requires special attention. Childhood diseases and disorders may be qualitatively and quantitatively different from their adult equivalents. This may affect either the benefit or the risk of therapies (or both) with a resulting impact on the risk/benefit balance.

The lack of reliable data in the paediatric population is associated with specific problems including: limited availability of safety data due to the lack of clinical trials in the paediatric population; under- or over-dosing in some age groups due to the lack of pharmacokinetics data or dose-finding studies; maturation, growth and development of the paediatric population susceptible to drug-induced growth and development disorders as well as to delayed ADRs not findable in adults.

Pediatricians should be aware that the use of off label drugs increases the risk of adverse reactions and they should also try to pay much more attention when they administer drug therapies to their patients, especially for those categories of drugs which have clinical studies supporting their safety and/or efficacy [1,2].

The World Health Organization defines adverse reactions as harmful and unintended responses to a drug and which occur with doses normally used in humans for prophylaxis, diagnosis or treatment of a disease or modifying a physiological function. It is a phenomenon which rarely is associated with a significant impact on the health system.

In 2001, according to a U.S. surveillance study, ADRs were the cause of 244.000 outpatient visits of children under 15 years of age. During an active surveillance of 63 U.S hospitals, the study of NEISS- CADES (National Electronic Surveillance System-Cooperative Adverse Event Surveillance project) reported that among the above mentioned age group the incidence of ADRs was equal to 2 out of 1000 people. Younger children are at greatest risk. Considering this, half of registered ADRs occur in people who are younger than 4 years of age and the risk of ADRs among children under 5 years of age is 4 times higher than those of children who attend schools (5.8/1000 compared to 1.1/1000). Even in Italy ADRs confirm their impact. According to a study based on a system of active monitoring of Paediatrics, about 15 out of 1000 children developed adverse reactions [1-6].

Despite a responsible attitude of paediatricians during the administration of medications and with an appropriateness of drug prescribing, the National Network of Pharmacovigilance (RNF) consistently underlines adverse drug reactions (ADRs) connected to medicines throughout the national territory [3-5].

Serious adverse reactions are rare and are generally not observed during the paediatric clinical trial program especially if there is a latent period before an onset or triggering such as a change in growth. For most medicines it is impossible to fully investigate rare adverse reactions prior authorization, as it is necessary to expose a large number of subjects to a medicinal product to elicit a reaction which occurs with a low probability in the target population.

Most of the ADRs, observed in pediatric prospective studies, mainly effect the skin (rash, urticaria) and the gastrointestinal system (diarrhea, nausea and vomiting), but we can also observe systemic reactions and reactions connected to the central nervous system. The drugs most frequently associated with adverse reactions are those most commonly used among young patients such as vaccines, antibiotics, antipyretics, non steroidal anti-inflammatory and specific products for colds and some drugs belonging to those classes used for the gastrointestinal tract and metabolism [3-5]. Although the overall incidence of data regarding the Italian situation is consistent when compared to other European nations, in some drugs we can be observe that significant differences also depend on national surgery practice.

## Off- Label drugs

Among the major factors that contribute to the occurrence of adverse reactions in children, the off-label use, or unlicensed, without authorization of use in children catches the eye. The limited availability of medicines specifically designed for the pediatric age is a reality with which many generations of pediatricians have learned to deal with. A reality which the practitioner has to face every time he or she is choosing a drug grouped with some evidence of efficacy and safety only in adults and not in smaller patients. Less than 15% of all drugs currently marketed and less than half of those specifically intended for children are operated on the basis of clinical trials which demonstrate specific features of risk-benefit balance in children [1,2,6-11]

The use of off-label or unlicensed is a therapeutic necessity and an opportunity where there would be, otherwise, a coverage of drug treatments. The highest percentages of off-label prescriptions are registered in complex diseases such as cancer, cardiovascular or renal diseases. In hospital wards corresponding to these therapeutic areas, like intensive care, 36-67% of children receive off-label prescriptions. In clinical practice, the outpatient prescription of off-label is 11-37% of the cases.

A systematic off-label use of drugs has been one of the main activities of the Working Group on "Medicines and Children " and of the AIFA Working Pediatric Group. An updated list of off-label drugs associated with the availability of scientific evidence was put together and sent to the relevant AIFA Technical Committee of Science (CTS).

The problem is that the use of off-label drugs exposes the child to a high risk of severe adverse reactions. There is a lack of specific formulations in extemporaneous preparations, for example when diluting concentrated solutions. The risk of medication errors is 3 times higher than those observed among adults. The incidence of error depends on the age of the patient, the therapeutic area, the setting and in most cases it is due to the need to adapt, in a very simplistic manner, an adult dose according to the difference in weight and body surface of a child. Quoting the joint WHO-UNICEF report of 2006: "Children are not small adults when taking a drug." The capacity of absorption, distribution, metabolism and elimination of a drug are very different between adults and children and they continue to change during the stage of development. The risks resulting from the administration of a drug that has not been tested and proven in the pediatric population may therefore be due to overdose (increase in adverse reactions), ineffectiveness of the drug (for dosing) and use of a formulation which is not appropriate [6-11].

A high percentage of the accesses to the emergency room for adverse reactions, after an ingestion of an overdose of medication, has been registered. The main causes of overdoses are: accidental ingestion of the drug for the lack of adult control, defective or inadequate packaging (for example, the lack of safety lock systems), the error in the preparation/dilution of a more concentrated drug. These also include the ingestion not being aware of the same active ingredient in drugs sold under a different name and a different indication.

One example that continues to reach the RNF reports of ADRs in children is of overdose of oxatomide. There are two different packages on the market with the same dosage form but at different concentrations (0.25% and 2.5%), which shows an increased risk of overdose linked to an incorrect administration [3-6].

## Clinical studies, evaluation and limitations

Before registration, children medication studies would be able to reduce at least some of the adverse reactions that are observed in young patients, especially those related to problems of dosage, overdose or poisoning. The pharmaceutical industry itself has little incentive to develop drugs and produce guidelines on doses for children because, apart from a few therapeutic areas, the potential market for pediatric medicines often offer marginal profits and are unlikely to cover the costs of a clinical trial. The studies are expensive and should be carried out separately for different age groups (newborns, infants, children, adolescents) because of the continuous metabolic changes and the maturation of the apparatuses.

In order to fill the void of studies and to improve knowledge about the efficacy profile of drugs in pediatrics, EMEA - according to Law 1901/2006, which became enforceable in 2007, as well as providing guidelines in order to conduct studies in Pediatrics (PIP, Pediatric Investigation Plane) - has established some incentives for pharmaceutical companies, including the extension of one year for the duration of the patent for those products used for experimentation on children.

Work is underway to highlight the fundamental need of medical treatment for children in order to identify old molecules, which could be revised and returned to pediatric use with a rational perspective, directed and implemented towards those areas which are still without proper treatment. This work has been started by the so-called Lists of Pediatric Needs developed by the EMEA. Priority has been established based on the severity of the disease, the lack of an alternative therapy and the existence of scientific data that has promoted the use of a molecule.

## Spontaneous reports

Pre-marketing trials are able to provide information about the benefits of drugs but do not manage to establish a safety profile. Therefore, spontaneous reporting of suspected ADRs become an important means to promote reasonable warning signs: from the description of a few cases, significant regulatory actions may derive in order to protect pediatric age groups (nasal decongestants on those banned below 12 aa, metoclopramide prohibited below 16aa., on eyedrops containing phenylephrine for mydriasis) [3-11].

Paediatricians' case reports mainly involve vaccines (exavalent, Anti-pneumococcal, anti-meningococcus, Trivalent measles/mumps/rubella) because they are used in most pediatric subjects or either because they involve the duty to report any type reaction including those which are expected not to be serious. Vaccines, which are then followed by frequency reports of pharmaceuticals used by children: antibiotics, antipyretics, specific products for colds and some belonging to those classes used for the gastrointestinal tract and metabolism [3-6].

The reports are verified and compared with data from reports presented in international networks EudraVigilance VigiMed Organization, but also on the basis of epidemiological studies and periodic drug safety data. In Italy, the activity of spontaneous reporting in children is very low: in recent years paediatric data-reporting has stood at around 1.6 to 1.8 percent, compared with 8% of total reporting [3-5].

Reasons may be different: 1. a non spread of a iatrogenic disease culture over the years 2. not having understood the real benefits that the spontaneous reporting system can create for the community in terms of reducing the risks of patients and saving resources 3. the lack of education, "mindset", "courage" of pediatricians as they tend "not to see" an injury caused, even though unintentionally, to their patients. In support of the above, there is the fact that, during the course of university studies and schools of specialization, inadequate attention is given to pediatric pharmacology, pharmacovigilance (FVG) and pathology of drugs, this causing methodological and behavioral uncertainty [1,2,11].

Other reasons can be the fear to report something already known and therefore considered of little value or exposure when speaking about off-label use of drugs; the lack of meaningful feedback on clinical practice; the perception that the compiling of report forms is complicated.

With regards to this, family pediatricians ask companies which produce software for children to simplify the procedure of reporting by inserting a specific link in their database program.

A major cause of underreporting by family pediatricians is determined by the behavior of families, when facing adverse reactions due to a non-rational use of drugs (often caused by the use of "Do it yourself" drugs), as they prefer to go directly to the Emergency Department after an ingestion of an overdose of medication (see Off-label drugs).

## Post-marketing studies and long term follow up (LTFU)

Even when conducted with a thorough and careful testing prior to the marketing of a drug, their characteristics are not always able to provide the information required in order to determine the safety profile of a drug used in the general population. This limited number of subjects includes and usually does not exceed 10,000, not to mention the adverse reactions (for example, with an incidence of 1 in 100,000 users) or to identify the real impact on public health. The short course of clinical trials does not disclose reactions with long latency, such as those related to chronic use. The patients selected are generally healthy and therefore have not been treated with other drugs.

Epidemiological post-marketing studies, directed towards the recovery of all the events which occur during monitoring, give much more reliable estimates of the risk of ADRs than those resulting from spontaneous reports [1,2,6-11].

"Risk management" programs promoted by industries are often under funded, therefore there is a need to improve drug monitoring programs by means of an "active" observation, this enabling the system to anticipate possible identifications of efficacy and safety problems. This would then help to plan appropriate actions in due time reducing risks for the community. The primary objective of a good FVG activity is the definition of the risk/benefit ratio. For a more precise and consistent verification of this report there are continuous investments in key inputs, and there is the contribution of a careful and continuous scientific evaluation of what are called "weak signals". Greater the transparency and simplification of procedures in the FVG field, the greater the hope of positive benefits in terms of public health and improved safety [1,2,6-11].

Another important aspect to be considered is the lack or the few studies which assess long-term ADRs in pediatrics probably because there are organizational and logistical difficulties (the cases refer to significant drop-outs in the studies carried out so far).

In order to complete the safety profile of a drug it is necessary to carry out post-marketing epidemiological studies aimed at a long-term recovery of all the events that occur during monitoring. In these studies a significant role can be played by the family pediatrician with the reporting of suspected ADRs and with the participation to active pharmacovigilance projects.

With regards to this, family pediatricians have organized a network of pediatric investigators (FIMP - Medicines for Children Research Network) spread throughout the country and involved exclusively in post-marketing studies and LTFU (Long Term Follow Up) studies according to the Law - DM 139/2001 (this Law expresses the possibility of family pediatricians to perform Phase 3 and Phase 4 in their clinical trials).

## Pro active FVG: challenges and opportunities for Family Paediatricians

Despite the increasing accuracy of testing, preclinical and clinical trials which are required during the development of any active substance for the purpose of ensuring their safety in clinical applications, some ADRs may be known in their qualitative aspect (type of side effect) and quantitative aspect (true incidence in the treated population) only after successful marketing and use in the population during a "normal" use and not in those selected for the clinical trials. When the drug is used in clinical practice in large unselected populations, epidemiological post-marketing studies are useful as they find their major confirmation in recalling all the events that occur during monitoring, with estimates of incidence of ADRs that can not be obtained by spontaneous reports.

From here the need to improve surveillance programs through the experience of use of drugs and resources of surveillance systems called "pro-active". Moving from the focus on a "defensive" regulation based on a type of surveillance of "passive" reactions to a now newer concept, FVG has to take into account the course that accompanies the whole life-cycle management of the drug. The primary objective of a good FVG activity is the definition of the risk/benefit. For a precise and consistent verification of this report, there are continuous investments in key inputs with the contribution of all the careful and continuous scientific evaluation of spontaneous reports. Having a proactive approach means to organize FP in terms of methods and resources so that the system is able to anticipate the possible identification of efficacy and safety problems in order to plan appropriate actions in due time reducing community risks [1,2,8,11].

From all these considerations it is clear that we should move in different directions. There is the need to awaken FPs to a culture of iatrogenic disease, to the reporting of ADRs and to the proper use of reporting forms. On the other hand there is a need for a greater culture of research in pediatric pharmacology leading towards an increasingly pro-active FVG. Moreover, we must be able to seize the opportunities independently from scientific research institutions (AIFA, Contract Department, FVG Regional funds) proposing safety studies on drugs for pediatric trials with short term follow-ups and particularly in the long run.

The organization of specific training courses should meet the following objectives: 1) promote the culture of iatrogenic disease in pediatrics 2) provide information on the benefit-risk profile of drugs 3) promote spontaneous reporting 4) improve the short and long term follow-up for a complete evaluation of ADRs.

A special FIMP working group, Pharmacovigilance Group: a) organizes training courses b) improves the Network of Pharmacovigilance in order to enable a synergy when working with the RNF and raising awareness to the alert among the FP c) provides a national Survey (drugs and/or pathology Registers) d) participates, when called for independent research, to evaluate the risk/benefit of drugs in pediatrics e) fosters close collaboration with the national regulatory activities through the participation in the AIFA Pediatric Working Group.

In this regard, the Group has already organized a Master Course "Pharmacovigilance in Pediatrics" which is part of a "pilot training and educational project". Another important event was a whole session in the II National Congress of Naples FIMP-2008 on "Medicines for children and Pharmacovigilance". In all Courses for FP-Investigators (according to the Law - DM 139/2001), which have been organized throughout the territory, specific ADRs sessions have been included. They all had the aim of raising the awareness of Family Paediatricians speaking about a culture of diseases caused by drugs.

## Future prospects for family pediatricians

A future path to follow in pediatrics is undoubtedly towards the synergy and coordination of all components of Pediatrics (universities, hospitals, territories) for the organization of research projects and post-marketing programs. The starting point must necessarily be cultural: an implementation of the culture of iatrogenic disease and a careful assessment of the significance of post-marketing studies by Family Paediatricians. Of course, all these supported by capacity building of the Network of FP-Investigators which is central to epidemiological studies-observational and DBPC-RCT studies on medicines for children. The creation of a specific database managed and coordinated by the FPs with medium and long term follow-up results becomes strategic and very important for a careful and correct evaluation of ADRs. Last but not least, what must also be mentioned, is the fact that with the FP there would be fewer drop-outs in LTFU if there is a taking charge of children and establishing a close relationship with families.

The ultimate goal is the coordination of the Italian network with national networks of other EU countries and then the becoming part of the EMEA "Network of the Networks'.

## Author Information

The author is President of the S.I.P (Italian Society of Paediatrics), the Head of FIMP- MCRN (Family Pediatricians -Medicines for Children Research Network) and Member of Pediatric Working Group AIFA (Italian Medicines Agency).
